# Fluorinated PLGA-PEG-Mannose Nanoparticles for Tumor-Associated Macrophage Detection by Optical Imaging and MRI

**DOI:** 10.3389/fmed.2021.712367

**Published:** 2021-08-27

**Authors:** Giorgia Zambito, Siyuan Deng, Joost Haeck, Natasa Gaspar, Uwe Himmelreich, Roberta Censi, Clemens Löwik, Piera Di Martino, Laura Mezzanotte

**Affiliations:** ^1^Department of Radiology and Nuclear Medicine, Erasmus Medical Center, Rotterdam, Netherlands; ^2^Department of Molecular Genetics, Erasmus Medical Center, Rotterdam, Netherlands; ^3^Medres Medical Research GmBH, Cologne, Germany; ^4^School of Pharmacy, University of Camerino, Camerino, Italy; ^5^Applied Molecular Imaging Facility of Erasmus MC (AMIE) Core Facility, Erasmus Medical Center, Rotterdam, Netherlands; ^6^Percuros B.V., Enschede, Netherlands; ^7^Biomedical MR Unit, Molecular Small Animal Imaging Center (MoSAIC), University of Leuven (KU Leuven), Leuven, Belgium

**Keywords:** cell tracking, perfluorocarbon, tumor-associated macrophage, contrast agent, ^19^F, magnetic resonance imaging, breast cancer

## Abstract

Tumor-associated macrophages (TAMs) promote cancer growth and metastasis, but their role in tumor development needs to be fully understood due to the dynamic changes of tumor microenvironment (TME). Here, we report an approach to visualize TAMs by optical imaging and by Fluorine-19 (^19^F) magnetic resonance imaging (MRI) that is largely applied to track immune cells *in vivo*. TAMs are targeted with PLGA-PEG-mannose nanoparticles (NPs) encapsulating perfluoro-15-crown-5-ether (PFCE) as MRI contrast agent. These particles are preferentially recognized and phagocytized by TAMs that overexpress the mannose receptor (MRC1/CD206). The PLGA-PEG-mannose NPs are not toxic and they were up-taken by macrophages as confirmed by *in vitro* confocal microscopy. At 48 h after intravenous injection of PLGA-PEG-mannose NPs, 4T1 xenograft mice were imaged and fluorine-19 nuclear magnetic resonance confirmed nanoparticle retention at the tumor site. Because of the lack of ^19^F background in the body, observed ^19^F signals are robust and exhibit an excellent degree of specificity. *In vivo* imaging of TAMs in the TME by ^19^F MRI opens the possibility for detection of cancer at earlier stage and for prompt therapeutic interventions in solid tumors.

## Introduction

Inflammation is one the major effect of cancer and it plays a pivotal role in cancer progression and metastasis (1). In healthy conditions, macrophages (Mϕ) exert pro-inflammatory and cytotoxic effect leading the immune response against tumor development (2). In solid tumors, tumor associated-macrophages (TAMs) are generally skewed away from the classical activation toward an alternative tumor promoting phenotype and becoming the major constituent of tumor malignancy (3, 4). Thus, presence of TAMs in the tumor microenvironment (TME) is correlated with increased tumor metastasis, angiogenesis, and tumor aggressiveness (5). In recent studies, histological sample of necrotic breast cancers have shown high tumor-associated macrophage infiltration correlating with unfortunate prognosis (6). Indeed, TAMs can efficiently enter the necrotic core of the breast cancer and still functioning in hypoxic-necrotic areas. In this regard, the ability to label and observe TAMs non-invasively and over the time can tremendously help to understand the temporal and spatial localization of this population in the TME (7).

Recently, the magnetic resonance imaging (MRI) technique has been used to image inflammation and to track immune cells *in vivo* with no need of radiation (8). In particular, perfluorocarbons (PFCs) are emerging as promising contrast agents for MRI cell tracking (9, 10). This is because, fluorine-based contrast agents are found only in traces in biological tissue meaning that the fluorine background is minimal and that the signal from exogenous fluorine is highly specific *in vivo* (10). Amongst PFCs, perfluoro-15-crown-5-ether (PFCE) is one of the most attractive MRI contrast agents because it is FDA approved in a form of emulsion and therefore it is not toxic (11). However, most of PFCs are not miscible with hydrophilic or hydrophobic solvents due to the strong carbon-fluorine covalent bond and strong electron withdrawing effects of fluorine. Thus, PFCs are typically prepared as lipid-based nano-emulsions with low toxicity and longer circulation time (12). However, nano-drops of PFCs show limited stability *in vivo* due to the low affinity amongst the PFCs, the continuous phase and the surfactant (7). In general, the physical structure of nano-emulsion may also restrict the combination with other functional molecules such as drugs, fluorescent tracker or surface ligand for specific targeting. To this purpose, biodegradable organic-based nanocarriers like liposomes, dendrimers, micelles, and polymeric NPs act as protector and provide a good stability of the payload (13). In this context, different strategies can be used for tumor targeting and tumor imaging (14). For instance, “Passively targeted” nanoparticles (NPs) exploit solely the enhanced permeability and retention (EPR) effect and allow to target cancer systemically. However, the circulation of passively targeted NPs is often prevented by main physiological barriers: the extravasation of the tumor vasculature especially for high-EPR tumors that reduces nanocarrier accumulation; the NPs clearance by mononuclear phagocytic system (MPS), sinusoidal cells of the liver and Kupffer cells (15). On the contrary, “actively targeted” nanoparticles can help to overcome such barriers and to deliver greater amount of payload to the desired compartment thanks to the functionalization of the polymeric surface. Amongst nanocarriers, poly-lactic-*co*-glycolic acid (PLGA) an FDA approved copolymer, is one of the most exploited system in pre-clinical research owed to its biodegradability, biosafety, biocompatibility, versatility in formulation and functionalization and long shelf-life (16, 17).

Herein, we have focused on ^19^F-based PLGA nanoparticles (NPs) to detect TAMs accumulation in humanized mice bearing breast cancer as tumor model (4T1 cells). To this purpose, PLGA NPs have been designed to encapsulate PFCE contrast agent and preserving its magnetic properties (18, 19). In addition, the polymeric shell of PLGA has been functionalized with polyethylene glycol (PEG) chains that enhance the plasmatic half-life of PLGA NPs and prevents the rapid opsonization by the mononuclear phagocyte system (MPS) for *in vivo* purposes. To actively target tumor-associated macrophages, the surface of PLGA-PEG nanoparticles has been also decorated with mannosamine ligand that is preferentially recognized and internalized by TAMs overexpressing mannose receptors (CD206) (20). In addition, fluorescein isothiocyanate (FITC) has been linked to the polymeric shell of the PLGA-PEG NPs allowing further *in vitro* and *ex vivo* validations. All in all, intravenously injected mannose- decorated ^19−^F based-PLGA-PEG NPs aim to enhance targeting of recruited tumor-associated macrophages in a humanized mouse model of breast cancer by ^19^F-MRI.

## Materials and Methods

### Materials

Unless stated, chemicals were purchase from Sigma Aldrich (Stenheim, Germany) and used as received. Poly (D,L-lactide-co-glycolide) (PLGA) copolymer (50/50, Resomer RG502H Mw 24,000-38,000) was purchased from Boehringer Ingelheim (Ingelheim am Rhein, Germany). Perfluoro-15-crown ether (PFCE) was provided by Exfluor Research Corporation (Texas, USA). Agilent Polystyrene calibration kit for GPC characterization was obtained from Agilent Technologies (Santa Clara, U.S.A.). Ultrapure water was produced in the laboratory according to a Milli-Q® system (Merck Millipore, Darmstadt, Germany).

### Proton Nuclear Magnetic Resonance Spectroscopy (^1^H-NMR)

Chemical structures and number-average molecular weight (Mn) of synthesized polymers were characterized by proton nuclear magnetic resonance spectroscopy (1H NMR, Varian Mercury plus 400, Crawley, UK) using CDCl3 or D2O as solvents. Chemical shifts were referred to the solvent peak (δ = 7.26 ppm for CDCl3, δ = 4.79 ppm for D2O).

### Gel Permeation Chromatography

Gel permeation chromatography (GPC) was employed to determine the weight average molecular weight (Mw), number average molecular weight (Mn) and the polydispersity index (PDI) of copolymers. GPC measurements were carried out by using a TSK gel G4000HHR column (Tosoh Bioscience, Tokyo, Japan), 7.8 mm ID × 30.0 cm L, pore size 5 μm. Polystyrenes of defined molecular weights ranging from 580 to 377,400 Da were used as calibration standards. The eluent was tetrahydrofuran (THF), the elution rate was 1.0 ml/min and the column temperature was 35°C. The samples were dissolved in THF at a concentration of 5 mg/ml.

### Fourier Transform Infrared Spectroscopy

Infrared spectra of the synthesized polymer were recorded by a Fourier transform infrared spectrophotometer (FT-IR, PerkinElmer, USA) at the wavelength range of 4,000-500 cm^−1^. All the spectra were recorded against a background of an air spectrum.

### Synthesis and Characterization of Polymers: PLGA-PEG-COOH, PLGA-PEG-FITC, and PLGA-PEG-Mannosamine

Carboxyl terminal groups of PLGA were activated and converted to PLGA-NHS for the subsequent conjugation with polyethylene glycol (PEG). Briefly, 2 g PLGA 503H polymer was dissolved in 10 ml anhydrous dichloromethane (DCM) followed by adding an excess of N-hydroxy succinimide (NHS, 46.0 mg, 0.4 mmol) and N,N'-dicyclohexylcarbodiimide (DCC, 82.5 mg, 0.4 mmol). The reaction was stirred at room temperature overnight, under the N2 atmosphere. To purify, PLGA-NHS was precipitated in diethyl ether and washed by cold mixture of diethyl ether and methanol three times to remove the residual NHS.


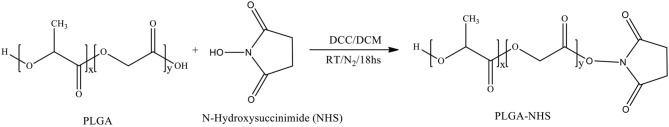


Chemical Synthesis Scheme of PLGA-NHS

**PLGA-PEG copolymers** with carboxyl terminal groups and amino terminal groups were synthesized by conjugated amino groups of NH_2_-PEG-COOH and NH2-PEG-NH_2_ correspondingly to the N-hydroxysuccinimide esters of resulting PLGA-NHS. In details, PLGA-NHS (500 mg, 0.015 mmol) was dissolved in 5 ml anhydrous DCM. Then NH_2_-PEG-COOH or NH2-PEG-NH_2_ (44.1 mg, 0.015 mmol) was added in the DCM solution with trimethylamine (TEA, 13.3 μl, 0.09 mmol) as catalyst. The reaction was processed at room temperature overnight, under N_2_ atmosphere. PLGA-PEG copolymer was precipitated with a cold mixture of diethyl ether and methanol and washed three times by the same solvents, then dried by desiccator under vacuum. number-average molecular weight (M_n_), the molecular weights (M_w_) and the polydispersity index (PDI) were characterized by gel permeation chromatography (GPC, TSK gel G4000HHR column (Tosoh Bioscience, Tokyo, Japan). Mn and chemical structures were determined by proton nuclear magnetic resonance spectroscopy (^1^H-NMR, arian Mercury plus 400, Crawley, UK).


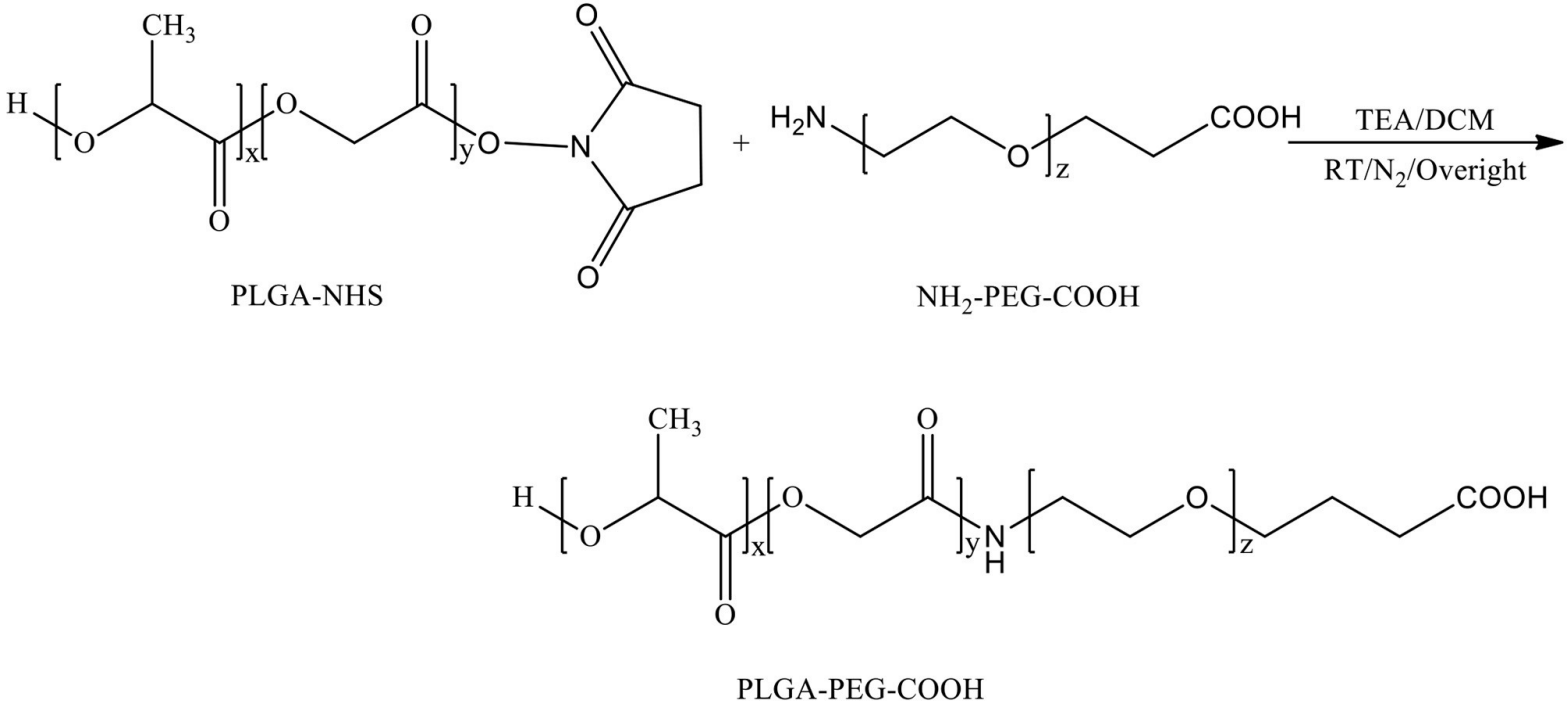


Chemical synthesis scheme of PLGA-PEG-COOH


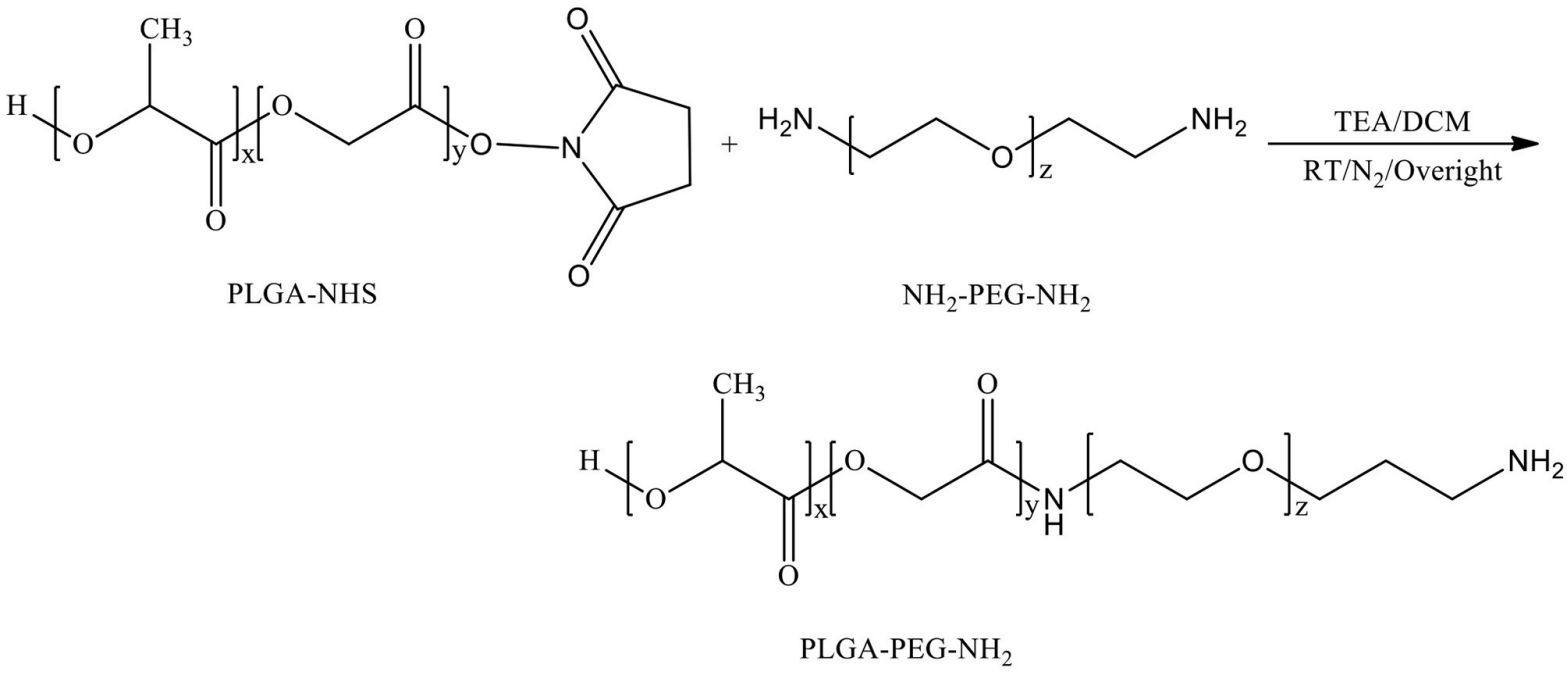


Chemical Synthesis Scheme of PLGA-PEG-NH_2_

**D-mannosamine** was covalently conjugated to the acid terminal groups of PLGA-PEG-COOH copolymer to yield PLGA-PEG-mannosamine copolymer. Briefly, the synthesized PLGA-PEG-COOH copolymers (200 mg, 0.006 mmol) were dissolved in 2.5 ml D-mannosamine solution in Dimethyl formamide (DMF) at a concentration of 0.025M. Then, 4-dimethylaminopyridine (DMAP, 7.3 mg, 0.06 mmol) and DCC (123.8 mg, 0.6 mmol) were added stepwise. The reaction mixture was stirred at room temperature overnight under nitrogen atmosphere. PLGA-PEG-mannosamine was precipitated in a cold mixture of diethyl ether and methanol, dried by desiccator under vacuum. Mn, Mw, and PDI were characterized by GPC, and chemical structures were determined by ^1^H-NMR spectroscopy.


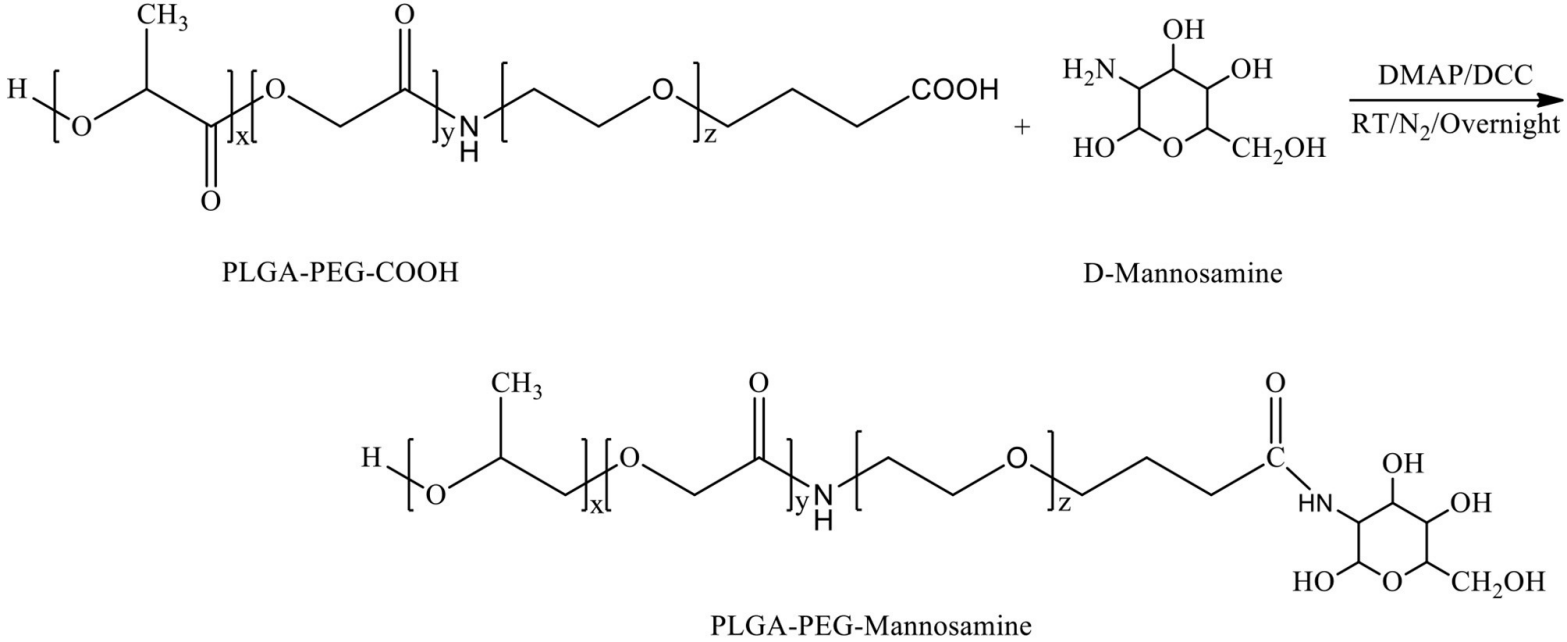


Chemical Synthesis Scheme of PLGA-PEG-Mannosamine

**Fluorescein isothiocyanate (FITC)** was conjugated to PLGA-PEG-NH_2_ to yield fluorescently labeled PLGA-PEG nanoparticles. FITC (4.21 mg, 0.011 mmol) and PLGA-PEG-NH_2_ (100 mg, 0.0027 mmol) were dissolved in 2.5 ml anhydrous dimethylsulfoxide (DMSO) at room temperature overnight. To purify, the reaction mixture was dialyzed against DMSO and water sequentially (Mw cutoff = 12-24 kDa), then isolated by lyophilization as a yellow powder. The FITC conjugation was characterized by measurement of fluorescence absorption at an excitation wavelength of 490 nm and an emission wavelength of 530 nm using Spectramax (iD3, Molecular Devices, USA). The FITC conjugation yield was calculated according to Formula 1.

### Nanoparticle Formulation

**Figure 1:**
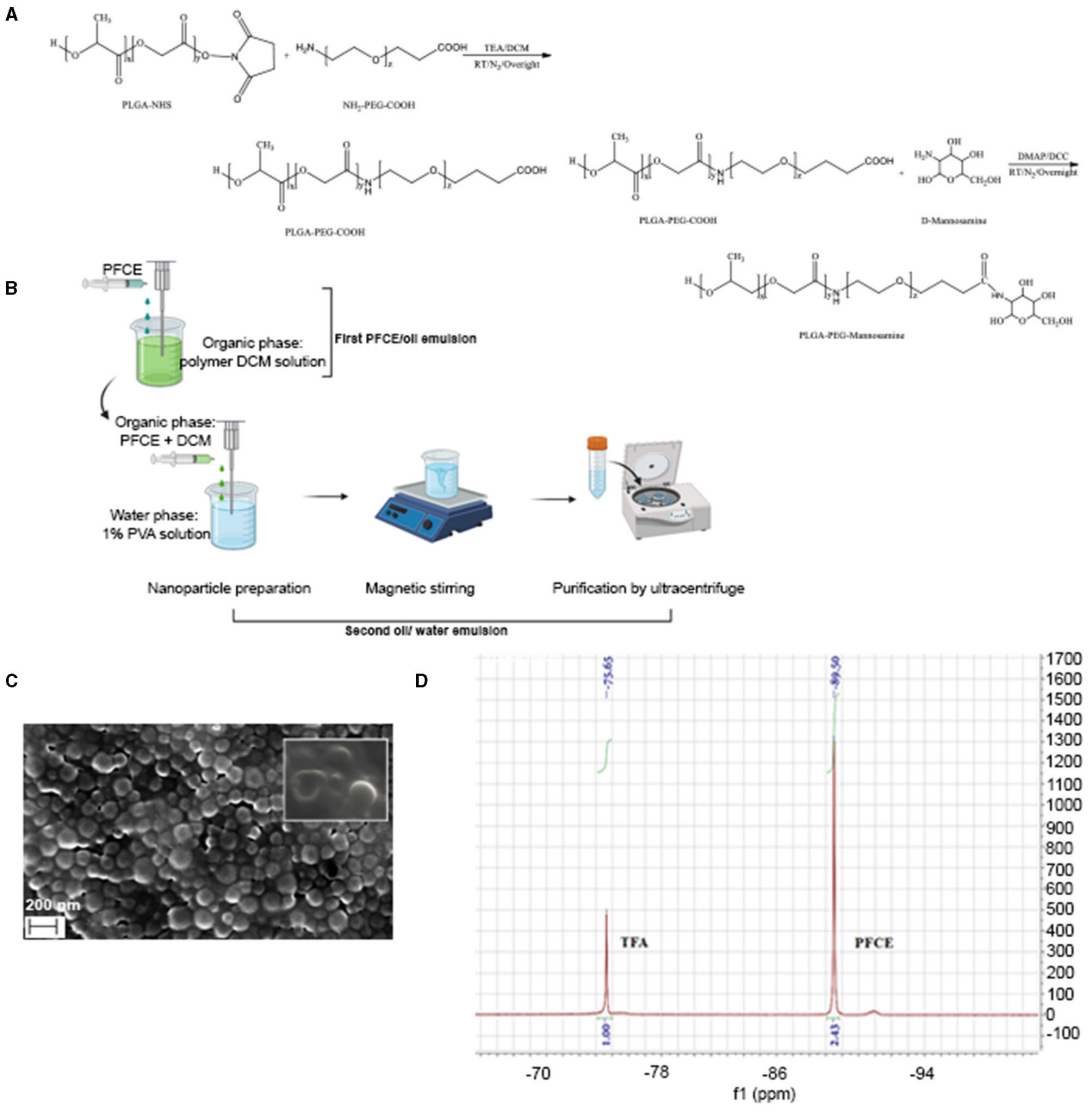
Chemical synthesis and characterization of PLGA-PEG nanoparticles. **(A)** Illustrative chemical synthesis of PLGA-PEG-COOH and PLGA-PEG-mannose copolymers. **(B)** Schematic illustration for PFCE/Oil/Water double-emulsion and solvent-evaporation method used to produce PLGA-PEG, PLGA-PEG-mannosamine, or PLGA-PEG-FITC nanoparticles. Scanning Electron Microscopy (SEM) of PLGA-PFCE nanoparticles. **(C)** Scanning electron microscopy (SEM) image of PFCE encapsulatin PLGA NPs. **(D)** ^19^F-NMR spectrum of PFCE encapsulated PLGA-PEG-FITC-mannose NPs in CDCl3 d in ppm: −75.65 (3F, CF_3_COOH); −89.50 (20F, C_10_F_20_O_5_).

PFCE encapsulated PLGA-PEG NPs were formulated by PFCE/O/W double emulsion solvent evaporation method using PLGA-PEG, PLGA-PEG-mannosamine, or PLGA-PEG-FITC to obtain NPs with different surface ligands (7). PFCE loaded PLGA or PLGA-PEG NPs formulation was described in Figure 1B. Briefly, the first emulsion was prepared by dropwise adding PFCE (890 μl) into 3 ml DCM containing 90 mg polymer along with homogenization (Ultra-Turrax T25, IKA-WERKE, Germany) at 3,000 rpm for 20 min at RT. Subsequently, the first emulsion was dropped into 18 ml of 1% w/v PVA water solution and homogenized (Ultra-Turrax® T25 digital, IKA, Staufen, Germany) in an ice bath at a speed of 13,500 rpm for 20 min. Then the emulsion was gently stirred at RT overnight for solvent evaporation and NPs solidification. NPs were isolated by centrifugation (High speed micro-centrifuge, D3024R, Scilogex, Rocky Hill, CT, USA) at 10,000 g for 30 min at 4°C, and washed three times by water to remove PVA. Afterwards, NPs were lyophilized (Freeze dryer, FreeZone, Labconco, Kansas City, MO, USA) by using 7% w/v sucrose as lyoprotectant and stored at −20°C. Empty NPs were prepared by suspended the first PFCE/O emulsion step, with only the second O/W emulsion evaporation in the same conditions as described.

### Size and Zeta Potential

Zeta potential, polydispersity index (PDI) and size of the nanoparticles were characterized by dynamic light scattering (DLS) at fixed at fixed 90° scattering angle at 25°C by Malvern Zetasizer 2000 (Malvern, UK). Suspensions were diluted in distilled water. Measurements were performed in triplicate at room temperature.

### Scanning Electron Microscopy

Nanoparticle morphology was determined by a field emission-scanning electron microscope (FE -SEM Zeiss Sigma 300, Zeiss, Germany). SEM sample stage was prepared by placing a double-sided adhesive carbon tape on an aluminum stub. One small drop of 1 mg/ml nanoparticle sample suspended in ultrapure water was placed on the sample stage and then dried at 37°C overnight. Subsequently, the dried sample was sputtered under vacuum with a chromium layer of approximately 100 Å thickness (Quorum Q150T ES, Quorum Technologies, UK) prior to analysis.

### Determination of PFCE Encapsulation Efficacy by ^19^F NMR

PFCE load content and encapsulation efficiency of PLGA-PEG, and PLGA-PEG-MN nanoparticles was determined by Fluorine-19 nuclear magnetic resonance spectroscopy (19F-NMR). Lyophilized nanoparticles were dissolved in CDCl_3_ containing 0.1 M trifluoroacetic acid (TFA) as internal standard. The amount of PFCE was calculated by the integration ratio between PFCE peak to TFA peak. Fluorine contents were calculated according to the **Formula 1** and **Formula 2**.

(1) $$\begin{array}{l} \text{PFCE load content }=\frac{PFCE\;volume\;loaded\;in\;nanoparticles}{Weight\;of\;nanoparticles} \end{array}$$

(2) $$\begin{array}{l} \text{PFCE encapsulation efficency \%}=\frac{PFCE\;encapsulated\;volume}{PFCE\;total\;volume\;}\;\times 100\%. \end{array}$$

### Cell Culture

Murine macrophage Raw 264.7 cell line and 4T1 cells (murine mammary carcinoma cells) purchased from (ATCC® TIB-71™) were cultured in complete DMEM medium (Sigma, St. Louis, Mo, USA) supplemented with 10% fetal bovine serum (FBS) and 1% of penicillin and streptomycin and incubated at 37°C with 5% CO_2_. When cell confluence reached around 80%, dead cells were washed away with PBS (Lonza) and live cells were detached by cell scraper. Cells were centrifuged and re-suspended with 8 ml of fresh DMEM medium. Cell counting was performed using BioRad TC20 cell counter.

### Cell Cytotoxicity and Uptake Assay of Polarized Macrophages

Cytotoxicity of targeted or untargeted PLGA or PLGA-PEG nanoparticles was tested for Raw 264.7 cells by Pierce LDH assay kit (Thermo Scientific) and following manufacturer's instructions. Cells were treated with nanoparticles at different concentrations ranging from 0 to 2.5 mg/ml and incubated for 24 h. For uptake assay, Raw 264.7 cells were first polarized for anti-tumorigenic (M1) or pro-tumorigenic (M2) phenotypes. M1 phenotype was made by incubating cells for 24 h with lipopolysaccharide (LPS) (100 ng/ml) and Interferon-gamma (IFN-γ) (50 ng/ml), both purchased from Sigma-Aldrich. M2 phenotype was made by incubating cells with Interleukin-4 (IL-4, Sigma-Aldrich) (20 ng/ml) for 24 h to obtain M2 highly expressing CD206 receptor. After polarization, cells were seeded in 24-well plates (8 × 10^4^ cells per well) and incubated with targeted or un-targeted PLGA-PEG nanoparticles (1 mg/ml). Incubation was performed for 1, 6, and 24 h at 37°C. After the incubation time, wells were gently washed with PBS to discard particles not up taken and green fluorescence of FITC was measured by selecting excitation wavelength at 490 nm an emission wavelength of 530 nm by Spectramax (iD3 series, Molecular Devices). Raw 264.7 cells not polarized (M0 phenotype) were used as control and all the tests were performed multiple times in triplicate.

### Fluorescence Microscopy

Internalization of PLGA nanoparticles targeted (PLGA-FITC-PEG-Mannose loaded with PFCE) or untargeted (PLGA-FITC-PEG loaded with PFCE) nanoparticles was confirmed by confocal microscopy. Raw 264.7 cells were seeded in a six well plate (80.000 cells per well). After cell attachment, cells were treated with targeted or untargeted nanoparticles (1 mg/ml) for 1 h. Wells were then washed three times and lysosomes were stained by deep red LysoTracker™ dye (Thermo Fisher Scientific) incubated for 20 min before cell fixation. Cells were then washed gently with PBS three times and fixated with 4% paraformaldehyde (PFA) for 20 min. After PBS wash, cell membrane was stained by PKH26 red fluorescent cell membrane label kit (Sigma-Aldrich, City, state) and nuclei were stained with Vectashield mounting-DAPI blue fluorescent dye (LSBio). Fluorescent NPs uptaken by Raw 264.7 cells were imaged by Leica SP5 confocal microscope equipped with Ar-He/Ne lasers (Leica Microsystems, Wetzlar, Germany). A 63x magnification with oil immersion objective (Carl Zeiss, Oberkochen, Germany) was used for cell imaging. Nanoparticles, cell membranes and lysosomes were visualized with respective channels at 488 nm (green), 561 nm (red), and 633 nm (deep-red).

### *In vitro* Fluorine-19 Magnetic Resonance Spectroscopy

Eppendorf tubes loaded with different concentrations of PFCE ranging from 5 to 100 μl were used to create a calibration curve. An MR 901 Discovery 7T magnet (Agilent Technologies, Santa Clara, CA, USA) with a preclinical front-end (GE Healthcare, Little Chalfont, UK) was used for MRS acquisition. The system is equipped with a gradient set with a maximum gradient strength of 300 mT m^−1^, a rise-time of 600 T m^−1^ s^−1^ and an inner diameter of 310 mm. For transmission and reception, an in-house-built dual tuned 1H/19F single channel surface coil with a diameter of 2 cm was used. The 19F MRS spectrum was recorded using a EchoSCI sequence (TR/TE = 1,250/15 ms, NEX = 128, FOV = 6 cm, slice thickness = 2,5 cm). MRS processing was performed in SAGE 7.6.2 (GE Healthcare, Little Chalfont, UK) on the MR 901 Discovery system. For processing of the data, time domain signals were apodized with a 10 Hz line broadening function, after which the signal was zerofilled to 4,096 points. Subsequently the time domain signal was Fourier transformed and the resulting spectrum was properly phased to show an absorption mode resonance line. 19F in the sample was quantified by reference to a standard curve, which was obtained by measuring a dilution series of PFCE with known ^19^F content.

### Mouse Model

BALB/c mice (6-8-week years old) were provided access to food and water *ad libitum* and were hosted in the animal facility at the Erasmus MC (Rotterdam, The Netherlands). All experiments were performed according to the guidelines for animal care of the Erasmus MC Animal Experiments Committee. For tumor mouse model, 8 x 10^4^ of LUC2 luciferase-expressing 4T1 breast cancer cells were injected subcutaneously in the left flank of the mice (*n* = 4 mice for each group). This cell line has been chosen because is a late state of breast cancer and exhibits necrosis. Tumor growth was measured by calipers and by bioluminescence imaging by IVIS spectrum imager (model, Perkin Elmer, city, state).

### *In vivo* Fluorine-19 Magnetic Resonance Spectroscopy

^1^H and ^19^F images were acquired 48 h after injection of 1 mg/ml of targeted (PLGA- PEG -FITC-Mannose loaded with PFCE) or untargeted (PLGA -PEG-FITC loaded with PFCE) nanoparticles by 7T MRI system (Bruker Biospin, city, Germany). All the subcutaneous breast tumors have a diameter ranging between ~0.6 and ~0.8 mm^3^ of diameter. *In vivo* imaging was done using a custom built dual ^1^H/^19^F coil for *in vivo* imaging. Mice (*n* = 4 for each group) were anesthetized using 1.5%. isoflurane (Isoflutek, Laboratorios Karizoo). Body temperature was monitored and regulated during imaging. Reference tube of known ^19^F concentration (7.01E + 19 ^19^F for PLGA-PEG-Mannose concentrated 1 mg/ml; and 4.95E + 19 ^19^F for PLGA-PEG nanoparticles) was placed alongside the mouse to optimize quantification of fluorine detected at the tumor site.

Magnetic resonance spectrometry was used to measure the 19F content per cell. The ^19^F MRS spectrum was recorded using a EchoSCI sequence (TR/TE = 1,250/15 ms, NEX = 128, FOV = 6 cm, slice thickness = 2.5 cm). MRS processing was performed in SAGE 7.6.2 (GE Healthcare, Little Chalfont, UK) on the MR 901 Discovery system. For processing of the data, time domain signals were apodized with a 10 Hz line broadening function, after which the signal was zerofilled to 4,096 points. Subsequently the time domain signal was Fourier transformed and the resulting spectrum was properly phased to show an absorption mode resonance line. ^19^F in the sample was quantified by reference to a standard curve, which was obtained by measuring a dilution series of PFCE with known ^19^F content.

### *Ex vivo* Determination of PFCE Encapsulation Efficacy by ^19^F NMR Spectroscopy

A 400 MHz Bruker Avance II NMR spectrometer (Bruker Biospin, Rheinstetten, Germany) was used to perform 19F NMR with a 5 mm broadband probe, which can operate at 376.5 MHz for *ex vivo* experiments. Excised organs (liver, lungs, and spleens and 4T1 subcutaneous tumors) were harvested and flash frozen by liquid nitrogen. The prepared sample (0.4 ml, mixed with D2O) was transferred to a 5 mm NMR tube (Wilmad, Vineland, NJ, USA). As a reference compound, 5-fluorocytosine (0.1 ml, 5 mM 19F concentration) was added to determine the chemical shift and 19F concentration for each sample. The pH value of the sample was confirmed to be around 7 when preparing the sample using Bromothymol blue indicator and the temperature was maintained at 37 C during the experiment. The acquisition parameters were as follows: frequency = 376.5 MHz, spectral width = 350 ppm, relaxation delay = 5 s, data points = 64 k. After phase and baseline correction of the acquired ^19^F NMR spectra using the Topspin software (Bruker Biospin, Rheinstetten, Germany), the ^19^F NMR signals were quantified relative to the 5-fluorocytosine signals (reference) by peak integration. The total ^19^F content of the excised organs (4T1 tumor, liver, spleen and lungs) was determined and the results were normalized to the tissue weight generating a signal expressed as a number of fluorine atoms per gram of tissue.

### Statistical Analysis

The data are presented as mean ± standard deviation (SD). *In vitro* and *in vivo* tests were performed using Graphpad 7 software and One-way ANOVA and *t*-test analysis of variance were used to analyze the differences between the groups. Significance was attributed when *P* < 0.001 (^*^) for *in vitro* tests and *P* < 0.05 (^*^) for *in vivo* tests.

## Results

### Synthesis and Characterization of Polymers

PLGA-PEG-NH-_2_, PLGA-PEG-COOH, PLGA-PEG-FITC and PLGA-PEG-mannose copolymers were successfully synthesized with a yield of ~60-85%. The synthesis of PLGA-PEG-mannose copolymer is shown in Figure 1A. Details of the polymer characteristics such as number-average molecular weight (M_n_), the molecular weights (M_w_) and the polydispersity index (PDI) are provided in Table 1. All the resulting copolymers presented PDI approximately of 1.4-1.7 with unimodal and symmetric peak in the GPC. This result confirmed the synthesized copolymer possessed narrow distribution of molecular weight.

**Table 1:** Mn, Mw, and PDI of PLGA-PEG and PLGA-PEG-mannose copolymers.

**Name**	**Mw^**a**^ (kDa)**	**Mn^**a**^ (kDa)**	**LA:GA^**b**^**	**PLGA:PEG^**b**^**	**PDI^**a**^**
PLGA-PEG-COOH	14	10	1:1	1:0.93	1.4
PLGA-PEG-mannose	9.2	7.3	1:0.95	1:0.82	1.3
PLGA-PEG-NH_2_	18.7	12.7	1:1	1:0.98	1.5
PLGA-PEG-FITC	–	–	1:1	1:0.92	–

a *Determined by GPC;*

b *Determined by ^1^H-NMR*.

The conjugation of mannosamine to PLGA-PEG copolymer was confirmed by by comparing ^1^H-NMR spectra of PLGA-PEG-mannose to those of PLGA-PEG-COOH (Supplementary Figure 1A) and D-mannosamine (Supplementary Figure 1B). The peaks at 1.58 and 5.22 ppm were characteristics of methyl groups and methine groups of glycolic acid (GA) segments, while the peak at 4.82 ppm was attributed to methylene group of lactic acid (LA) segments, which were both contributed by PLGA chains (Supplementary Figure 1C). The peak observed at 3.64 ppm corresponded to methylene groups of PEG segment. The integration ratio between the characteristic peaks of PEG and PLGA chains reveals that PEG was chemically conjugated on PLGA with mole ratio around 1:1. The peaks of mannosamine overlapped with the peak of PEG (3.62 ppm), therefore only one small peak at 4.11 ppm was detected and attributed to mannosamine (21).

FT-IR analysis was carried out to further confirm the amide bond formed between PLGA and PEG segments, and also the chemical conjugation of mannosamine onto PLGA-PEG-COOH copolymer. FT-IR spectra of PLGA-PEG-COOH and PLGA-PEG-Mannosamine copolymers were displayed in Supplementary Figure 2. The absorption peaks at 1,630 and 1,510 cm^−1^ were assigned to the C=O and N-H bonds, respectively, which reveals the successful conjugation via amide linkage between PLGA and PEG segment of the PLGA-PEG copolymer (22). Compared with the spectrum of PLGA-PEG-COOH copolymer, the one corresponding to PLGA-PEG-mannosamine presents new peaks at 3,264, 2,917, 2,851 cm^−1^ attributed to the stretching vibrations of the O-H and the C-H bonds of methylene and methyne groups, respectively, which were contributed by mannosamine. This result demonstrated that the mannosamine was efficiently conjugated onto the PLGA-PEG-COOH copolymer. The FITC conjugation molar ratio of PLGA-PEG-FITC copolymer was 85% measured by the fluorescence absorption according to standard curve build by pure FITC solution.

### Formulation and Characterization of PLGA-PEG Nanoparticles

Formulation of PFCE loaded PLGA-PEG nanoparticles (NPs) is illustrated in Figure 1B. NPs showed a mean diameter in the range of ~239 and ~345 nm depending on nanoparticle formulation. PFCE encapsulated nanoparticles showed a slightly higher diameter of ~50 nm compared with the empty ones. All the particles were also monodispersed presenting a low polydispersity index (PDI). Additionally, all the nanoparticles displayed negative zeta potentials due to the existence of terminal carboxyl groups in the PLGA polymer that is in the deprotonated form at physiological pH (23). Indeed, zeta potential values were between −31 and −17 mV (Table 2). Mannosamine decorated PLGA-PEG NPs presented a less negative zeta potential compared with the other NPs without mannosamine due to the partial neutralization of the negative charges by mannosamine.

**Table 2:** Particle Size, PDI, and Zeta potential of PLGA-PEG nanoparticles.

**Nanoparticles**	**Load**	**Particle size (nm)**	**PDI^**[iii]**^**	**Zeta potential (mV)**
PLGA^[i]^ –PEG	-	258 ± 10	0.28 ±0.01	−22.3 ± 0.6
PLGA^[i]^ –PEG	+PFCE^[ii]^	371 ± 8	0.23 ± 0.03	−26.3 ± 0.4
PLGA^[i]^ -PEG-FITC	-	299 ± 12	0.36 ± 0.05	−26.8 ± 0.4
PLGA^[i]^ -PEG-FITC	+PFCE^[ii]^	345 ± 8	0.20 ± 0.01	−24.2 ± 0.2
PLGA^[i]^ -PEG-mannose	-	199 ± 3	0.10 ± 0.03	−12.9 ± 0.6
PLGA^[i]^ -PEG-mannose	+PFCE^[ii]^	386 ± 3	0.23 ± 0.00	−17.9 ± 0.1
PLGA^[i]^ -PEG-mannose-FITC	-	222 ± 4	0.22 ± 0.09	−24.2 ± 2.0
PLGA^[i]^ -PEG-mannose-FITC	+PFCE^[ii]^	318 ± 4	0.10 ± 0.02	−19.6 ± 0.4

[i] * PLGA, poly(lactide-co-glycolide)*.

[ii] *Perfluoro-15-crown-5-ether*.

[iii] *Polydispersity index*.

Nanoparticles (NPs) were observed by scanning electron microscopy (SEM) where images confirmed that the PLGA-PFCE nanoparticles had a relatively uniform diameter of ~ 200 nm with a spherical shape and smooth surface (Figure 1C).

In some cases, nanoparticles showed dented appearance supposedly due to the phase separation of PFCE from nanoparticles during the analysis (24). This finding is therefore an indirect effect of the presence of PFCE within nanoparticles.

As depicted in Figure 1D, two peaks were highlighted in the spectrum at δ = −75.65 ppm for fluorine atoms of TFA and at δ = −89.50 ppm for fluorine atoms of PFCE. The amount of PFCE encapsulated was calculated from the integration ratio between PFCE peak and TFA peak. PLGA-PEG and PLGA-PEG-mannose nanoparticles had a comparable fluorine encapsulation efficiency: 65.2% for PLGA-PEG, 67.1% for PLGA-PEG-mannose, and similar load content (6.45 μl/mg. for PLGA-PEG and 6.64 μl/mg for PLGA-PEG-mannose). Quantification and encapsulation efficiency are outlined in Table 3.

**Table 3:** PFCE load content and PFCE encapsulation efficiency PLGA-PEG nanoparticles characterized by *in vitro* ^19^F-NMR.

	**Name**	**Number of F-atoms**	**Load content (μl/mg)**	**Encapsulation efficiency (%)**
^19^F-NMR ^[i]^	PLGA-PEG-FITC- PFCE-	3.14 E + 20	6.45	65.2
	PLGA-PEG-mannose -FITC-PFCE	2.82 E + 20	6.64	67.1

[i] *Values are calculated for 1 mg of NPs dissolved in CDCl_3_ solvent before the analysis at ^19^F-NMR*.

### Cell Cytotoxicity and NPs Uptake Assay of Polarized Macrophages

**Figure 2:**
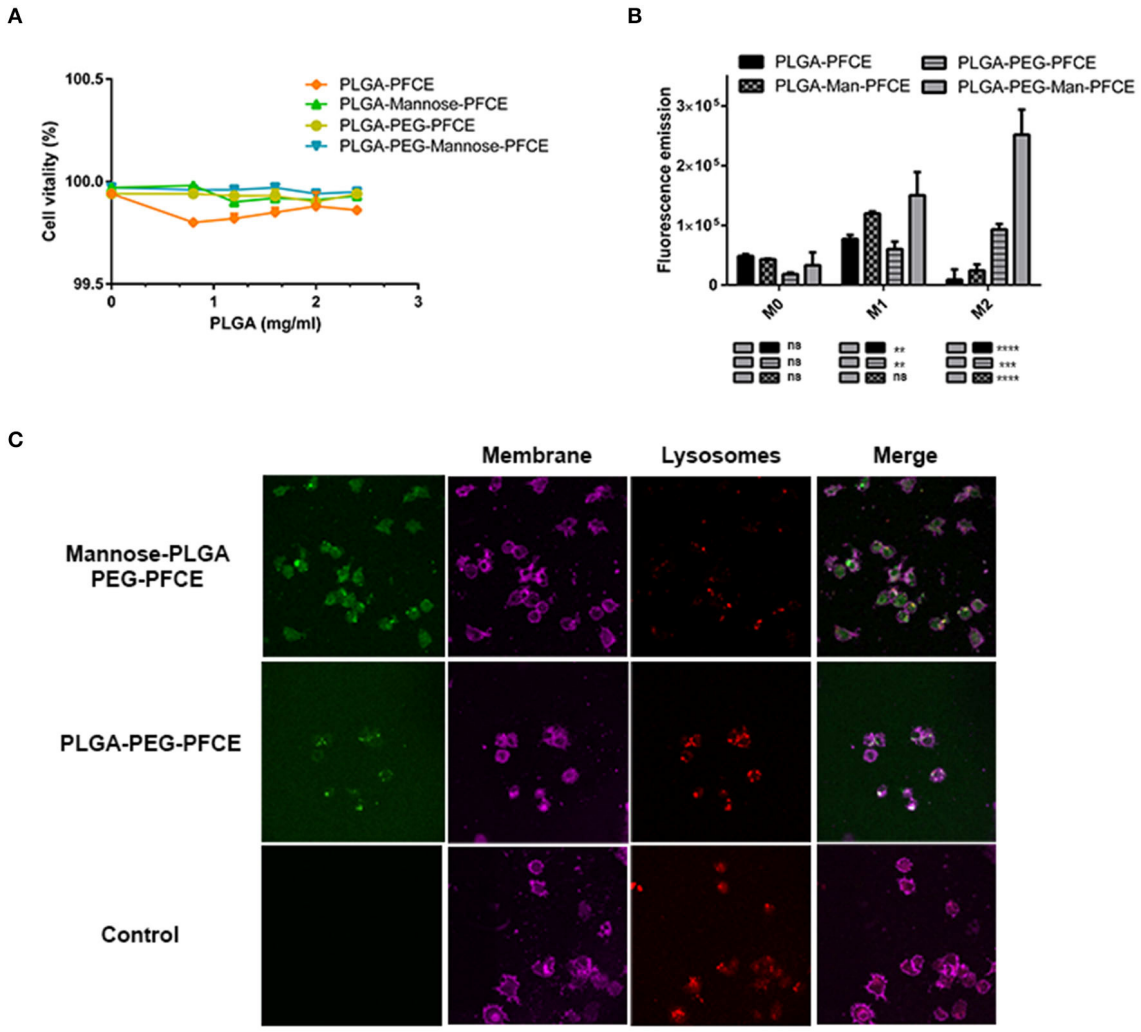
Characterization of nanoparticles *in vitro*. **(A)** Cytotoxicity tests for Raw264.7 macrophage cells treated for 24 h with PLGA-PFCE, PLGA-PFCE-Mannose, PLGA-PEG-PFCE, and PLGA-PEG-mannose-PFCE nanoparticles (final concentration 1 mg/ml). **(B)** Uptake efficiency of Raw264.7 macrophage cells polarized in M0 (negative control), M1 (pro-inflammatory), M2 (anti-inflammatory) phenotype and treated for 1 h with PLGA-PFCE, PLGA-PFCE-mannose, PLGA-PEG-PFCE and PLGA-PEG-mannose-PFCE nanoparticles (final concentration 1 mg/ml). **(C)** Confocal images of polarized M2-polarized Raw264.7 macrophages treated for 1 h with mannose (top) and untargeted (middle) PLGA-PEG NPs. Negative control for PLGA-PEG nanoparticles is shown in the bottom panels. Nanoparticles are shown in green color (FITC labeled); cell membrane is shown in magenta color; lysosomes are shown in deep-red color.

Initial studies were performed to assess cell viability when M2-like Raw264.7 macrophages were treated with targeted or untargeted PLGA nanoparticles. Cytotoxicity assay was performed incubating macrophages with different concentration of nanoparticles ranged between 0 and 2.5 mg/ml and incubated for 24 h at 37°C. Around 100% of cells were not affected by the treatment and all type of nanoparticles were well-tolerated (Figure 2A).

To test if fluorescent targeted or untargeted nanoparticles were preferentially up-taken by macrophages with M2-like phenotype, we first polarized Raw264.7 macrophages into M1-like macrophages, M2-like macrophages, and unpolarized macrophages (M0-like phenotype). Later, cells were incubated with targeted and untargeted PEGylation or not, FITC-PLGA nanoparticles (1 mg/ml) for 6 h. Figure 2B shows that targeted and PEGylated PLGA nanoparticles were preferentially up-taken by M2-like polarized macrophages. Particularly for the M0-like phenotype, not significant differences were highlighted by the uptake of the different nanoparticles. For the M1-like phenotype group, targeted-PEG-PLGA NPs particles had greater uptake (~1.20-fold) than un-PEGylated-mannose NPs and also higher uptake than untargeted PEGylated particles (~1.7-fold). For M2-like phenotype group, targeted-PEG-PLGA NPs have roughly a 2.5-fold higher uptake compared to untargeted-pegylated nanoparticles and ~eight-fold higher than un-PEGylated-mannose NPs. Statistical analysis was performed with one-way ANOVA, for triplicate samples and significance attributed when *P* < 0.001. Taken together, PLGA-PEG-mannose nanoparticles resulted in the highest uptake by M2-like macrophages compared to M0-like and M1-like macrophages, suggesting that both PEGylation and mannose ligand stimulate the cellular uptake.

To confirm cellular internalization of NPs, M0, M1 and M2-like Raw 264.7 macrophages were treated with targeted and untargeted nanoparticles conjugated or not with PEG (1 mg/ml). Confocal images of labeled polarized macrophages are shown in Figure 2C, where PLGA-PEG nanoparticles conjugated with FITC were stained in green, cell membrane in magenta, and lysosomes in red.

### *In vivo* Fluorine-19 Nuclear Magnetic Resonance

**Figure 3:**
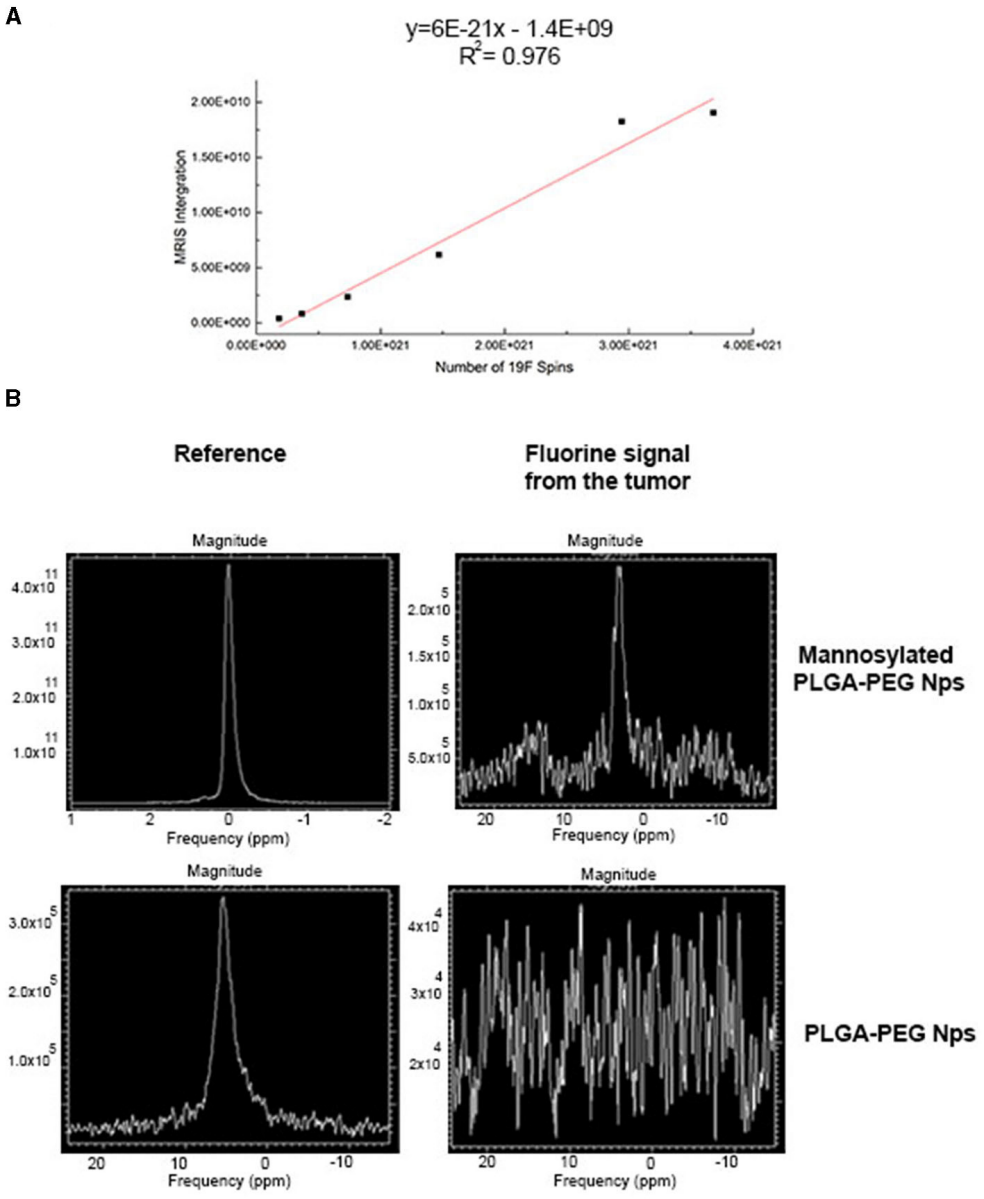
PFCE magnetic resonance measurement by ^19^F-MRI spectroscopy (MRS). **(A)** Standard curve of pure PFCE contrast agents measured at different dilution volumes ranging from 5 and 100 μl. **(B)** ^19^F-MRI spectrum of PFCE detected from 4T1tumor-bearing mice and treated with PLGA-PEG-mannose nanoparticles (top graph) and with PLGA-PEG nanoparticles (bottom graph). Respective external reference tubes (left) were used to set up image acquisition methods and for PFCE measurements at the tumor site.

In order to calculate the amount of ^19^F encapsulated in targeted and untargeted PLGA-PEG NPs, a curve of reference was built using different dilutions of pure ^19^F ranged between 5 and 100 μl (Figure 3A). *In vitro* quantification of ^19^F spins encapsulated in 1 mg/ml of targeted and untargeted PLGA-PEG NPs revealed an adequate number of ^19^F-atoms for further *in vivo* evaluations. In particular 7.01E + 19 ^19^F were detected for PLGA-PEG nanoparticles and 4.95E + 19 ^19^F were detected for PLGA-PEG-mannose nanoparticles.

4T1-breast xenograft mice (*n* = 4 per group) received 1 mg/ml of targeted or untargeted-PLGA-PEG nanoparticles intravenously (200 μl of suspension in PBS). ^19^F signals from the tumor site were quantified 48 h after NPs injection by MRS. A spectrum of ^19^F signal was successfully measured from the tumor area of mice injected with targeted PLGA nanoparticle by 7T MRI as shown in Figure 3B (top). As for untargeted-PLGA-PEG nanoparticles, the signal-to-noise ratio measured from the tumor site was low and fluorine quantification was not possible. This suggests that the targeted-PLGA-PEG nanoparticles have a more efficient accumulation at the tumor site compared to the untargeted-PLGA-PEG nanoparticles. Notably, preliminary data from *ex vivo* ^19^F NMR suggests that ^19^F signal detected in the tumors after treatment with targeted-PLGA-PEG NPs was four-fold greater compared to untargeted-PLGA-PEG NPs (Supplementary Figure 3).

## Discussion

In this study, we have assessed PLGA-PEG NPs decorated with mannose ligand for TAMs detection by ^19^F-MRI. This approach has exploited the use of different types of PLGA nanoparticles that are not toxic, stable and by definition more resistant to mechanical stress. In addition, polymeric nanoparticles of PLGA offer the advantage to be further functionalized with target ligands. PEGylation and mannosylation show an influence in circulation and cellular uptake of nanoparticles. Actually, first of all, PEGylation of PLGA nanoparticles protects them from complement activation (i.e., opsonization) with longer circulation in the blood stream, with the consequence of an improved opportunity for the drug to be released to the target site.

Secondly, mannosylation can act as cellular membrane-docking ligand allowing for nanoparticle internalization in mannose-expressing macrophages especially the M2 macrophages due to overexpression of mannose receptor. Thus, mannose can be used for intracellular delivery of relevant payloads (20, 25, 26).

Here, we decided to encapsulate the PFCE perfluorocarbon as contrast agent for ^19^F-MRI. PLGA nanoparticles used as carrier ensure PFCE stability for long storage, allowing for lyophilize, solubilize in suspensions and freeze the particles. In our study, we could produce PLGA nanoparticles of narrow size the distribution and a size approximately between 330 and 390 nm irrespective to the nanoparticle formulation (Table 2). SEM images for PLGA-PFCE NPs confirmed rounded and smoothed surface of nanoparticles. However, PLGA-PEG NPs did not provide resolved photos due to the interference of PVA surfactant with the analysis. Thus, images of NPs obtained by TEM would provide more accurate analysis for size and shape of PLGA-PEG NPs. NPs showed minimal toxicity *in vitro* when incubated with macrophages also for higher concentration like 2.5 mg (Figure 1A). However, it has been shown that vitality of cells is not affected if treated with 20 mg/million cells of PLGA nanoparticles (27). The ^19^F-payload of particles is similar amongst the different groups as demonstrated by ^19^F-MRS and ^19^F-NMR analyses. All the PFCE-nanoparticles were also able to target TAMs and be internalized by them especially if they were PEGylated and mannosylated (Figures 2B,C).

Finally, PFCE used for *in vivo* studies is known to be non-toxic in biological systems. Finally, PFCE used for *in vivo* studies is known to be non-toxic in biological systems. However, ^19^F-based cell tracking suffers from detection sensitivity and in general thousands of cells per voxel are required for detection of labeled cells (28). When injection of targeted-PEG-PLGA nanoparticles, fluorine signals were detected 48 h after injections. We observed higher liver retention of nanoparticles *in vivo* most probably due to the continuous uptake by liver like Kupffer cells, liver sinusoidal endothelial cells (LSECs), and hepatic stellate cells (HSCs) (29). This might be overcome by treating mice with clodronate liposomes before NPs injection and blocking non-specific uptake by Kupffer macrophages in the liver and increasing ^19^F-signal due to the greater retention of targeted-PLGA NPs in the tumor site (30). Recently, it has also been demonstrated that an antifouling-polymer coatings may block non-specific uptake of nanoparticles by liver (31). For untargeted PLGA-nanoparticles, the ^19^F signals in the tumor did not exceed the background noise arising from the surrounding organs. For targeted PLGA-nanoparticles in the tumor site, the ^19^F signals in the tumor had a weak intensity when measured by MRI *in vivo*, but the concentration of the PFCE could be successfully quantified by ^19^F MRS. Furthermore, *ex vivo* ^19^F NMR data confirmed higher retention of ^19^F signals at the tumor site after injection of targeted-PLGA-PEG NPs compared to injected untargeted-PLGA-PEG NPs (Supplementary Figure 3). We believe that higher magnetic field strengths or more sophisticated detectors might compensate with the sensitivity of the detection allowing to measure fewer amount of fluorine in the tumor. Altogether the results presented in the study prove the efficacy of delivering PLGA-PEG-mannose nanoparticles by TAMs reaching the tumor site *in vivo*. Future studies will focus on accumulation of functionalized PEGylated-nanoparticles delivered by TAMs as a function of tumor growth stage and as a function of the trafficking and timing of TAMs *in vivo*.

## Data Availability Statement

The original contributions presented in the study are included in the article/Supplementary Material, further inquiries can be directed to the corresponding author/s.

## Ethics Statement

The animal study was reviewed and approved by Erasmus MC Animal Experiments Committee (Animal work protocol number 17-867-53).

## Author Contributions

GZ, SD, LM, and PD designed the experiments. GZ, SD, and UH performed the experiments and analysed the data. GZ, SD, and LM wrote the manuscript. JH provided expertise on MRI measurements. GZ, SD, NG, RC, PD, CL, UH, and LM critically revised the manuscript for important intellectual content. All authors have read and agreed to the published version of the manuscript.

## Conflict of Interest

GZ was employed by Medres Medical Research GmBH (Cologne, Germany). NG is employed by Percuros B.V. (Enschede, The Netherlands). The remaining authors declare that the research was conducted in the absence of any commercial or financial relationships that could be construed as a potential conflict of interest.

## Publisher's Note

All claims expressed in this article are solely those of the authors and do not necessarily represent those of their affiliated organizations, or those of the publisher, the editors and the reviewers. Any product that may be evaluated in this article, or claim that may be made by its manufacturer, is not guaranteed or endorsed by the publisher.
