# Supplementary material for: Fluorinated PLGA-PEG-Mannose Nanoparticles for Tumor-Associated Macrophage Detection by Optical Imaging and MRI
Source: Front Med (Lausanne). 2021 Aug 27;8:712367. doi: 10.3389/fmed.2021.712367 (PMC8429784; doi:10.3389/fmed.2021.712367)
Supplement: Supplementary file 1 [file Data_Sheet_1.DOCX]

Fluorinated Mannose-PLGA-PEG nanoparticles for tumor-associated macrophage detection by optical imaging and MRI.

**Giorgia Zambito^1,2,3^** **^†^, Siyuan Deng ^4†^, Joost Haeck^5^, Natasa Gaspar^1,2,6^, Roberta Censi^4^, Clemens Löwik^1,2^, Uwe Himmelreich^7^, Piera Di Martino^4^ ^*^, Laura Mezzanotte ^1,2^***

**Supplementary data**

**Supplementary Figure 1a.** ^1^H-NMR spectrum of PLGA-PEG-mannosamine polymer in CDCl_3_, δ in ppm: 1.58 (3H, -O-CH(CH_3_)-);3.64 (4H,-CH_2_-CH_2_-O-) 4.1-4.2 (protons of conjugated mannosamine); 4.82 (2H, -O-CH_2_-C(=O)-);5.21 (-O-CH(CH_3_)-).


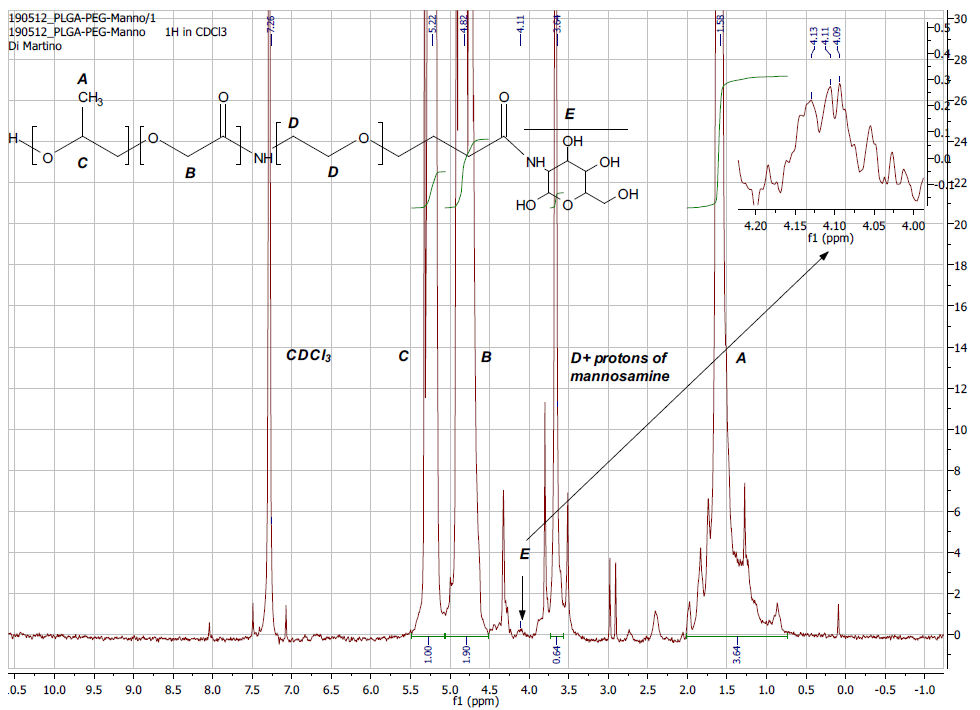


**
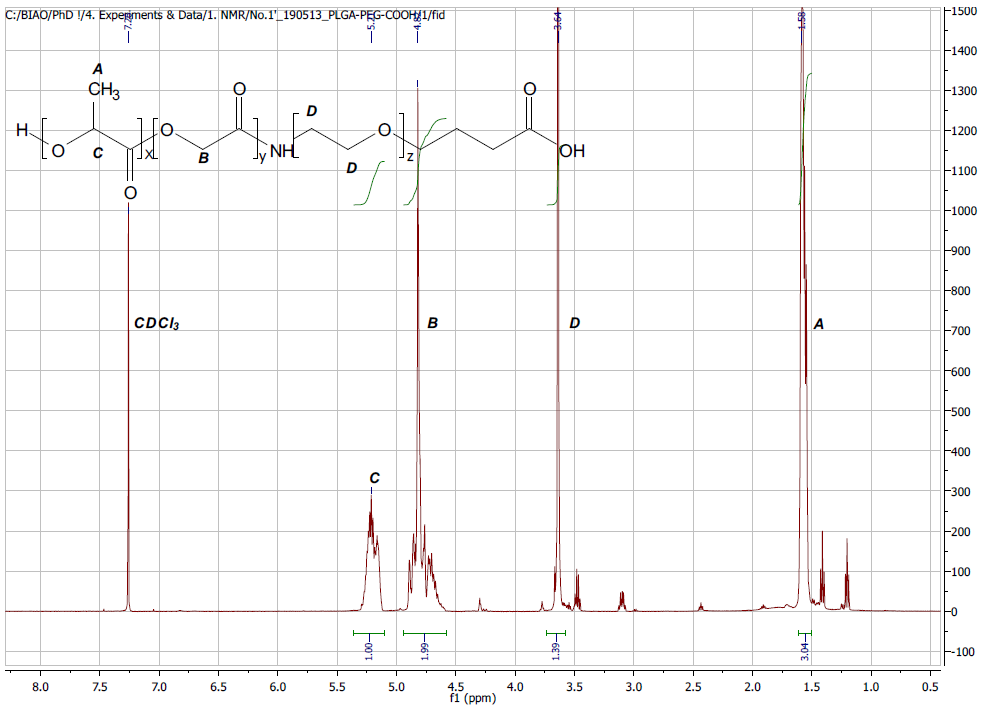
 Supplementary Figure 1b.** ^1^H-NMR spectrum of PLGA-PEG polymer in CDCl_3_, δ in ppm: 1.58 (3H, -O-CH(CH_3_)-); 4.82 (2H, -O-CH_2_-C(=O)-);5.21 (-O-CH(CH_3_)-).

**
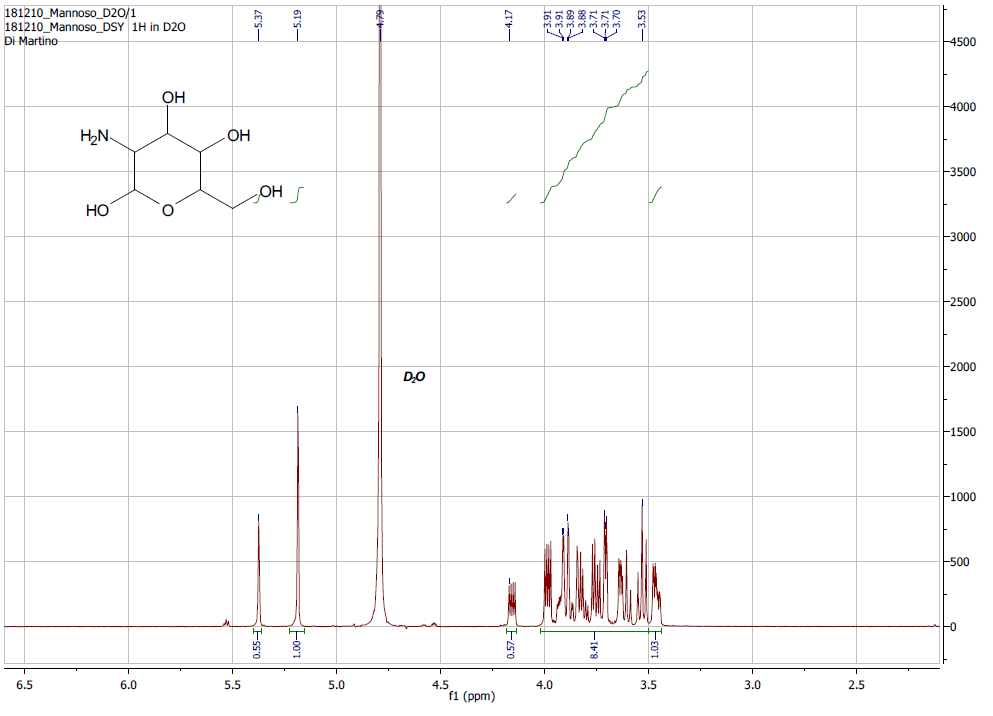
 Supplementary Figure 1c**. ^1^H-NMR spectrum of Mannosamine in D_2_O, δ in ppm.


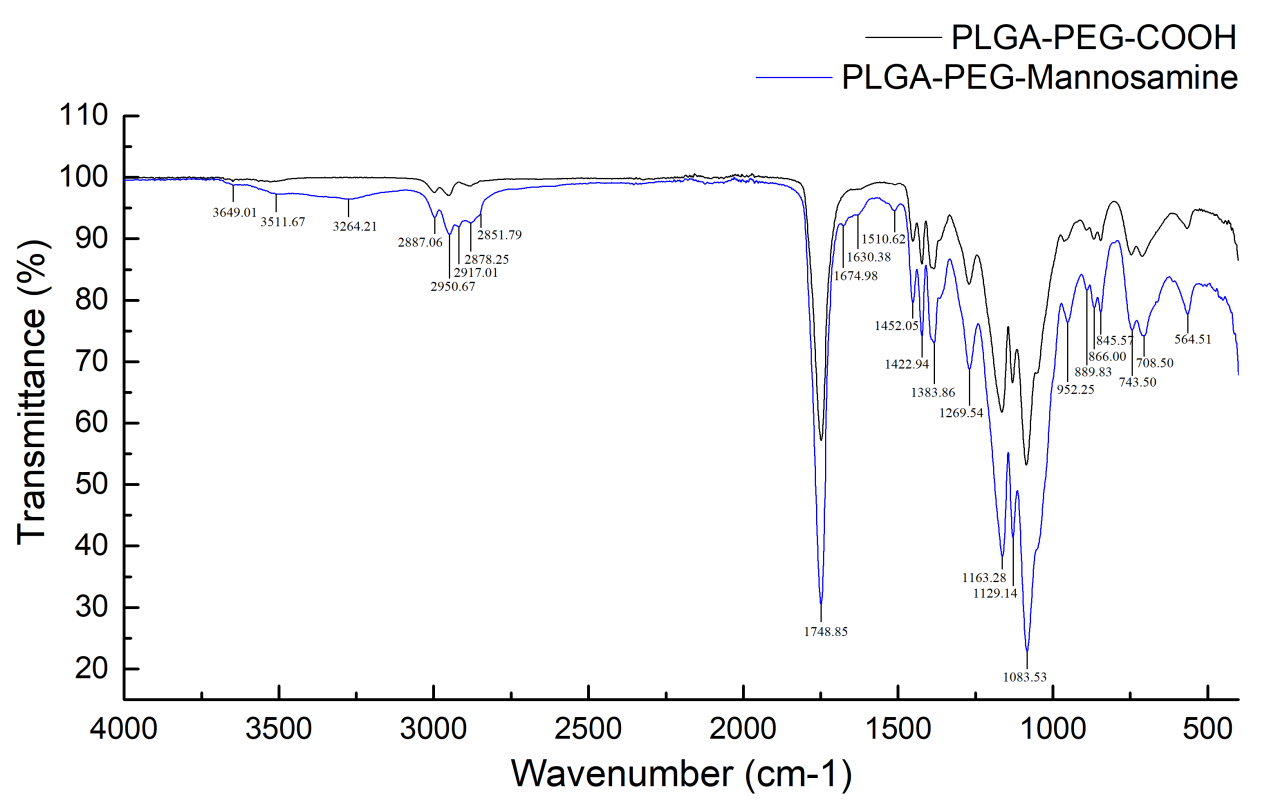


**Supplementary Figure 2**. FT-IR spectra of PLGA-PEG-COOH and PLGA-PEG-mannosamine copolymers.

**

**

**Supplementary Figure 3**. **Ex vivo ^19^F signals by NMR spectroscopy**. The total ^19^F content of the excised organs (tumor, liver, spleen and lungs) was determined and the results were normalized to the tissue weight generating a signal expressed as a number of fluorine atoms per gram of tissue. Technical details can be found in materials and method section.
